# Extracellular volume fraction mapping in the myocardium, part 2: initial clinical experience

**DOI:** 10.1186/1532-429X-14-64

**Published:** 2012-09-11

**Authors:** Peter Kellman, Joel R Wilson, Hui Xue, W Patricia Bandettini, Sujata M Shanbhag, Kirk M Druey, Martin Ugander, Andrew E Arai

**Affiliations:** 1 National Heart, Lung, and Blood Institute, National Institutes of Health, Bethesda, MD, USA; 2 Siemens Corporate Research, Princeton, NJ, USA; 3 National Institute of Allergy and Infectious Diseases, National Institutes of Health, Bethesda, MD, USA; 4 Department of Clinical Physiology, Karolinska Institute and Karolinska, University Hospital, Stockholm, Sweden

**Keywords:** Fibrosis, Edema, Gadolinium, Myocardial infarction, Hypertrophic cardiomyopathy, Dilated cardiomyopathy, Myocarditis, Systemic capillary leak syndrome

## Abstract

**Background:**

Diffuse myocardial fibrosis, and to a lesser extent global myocardial edema, are important processes in heart disease which are difficult to assess or quantify with cardiovascular magnetic resonance (CMR) using conventional late gadolinium enhancement (LGE) or T1-mapping. Measurement of the myocardial extracellular volume fraction (ECV) circumvents factors that confound T1-weighted images or T1-maps. We hypothesized that quantitative assessment of myocardial ECV would be clinically useful for detecting both focal and diffuse myocardial abnormalities in a variety of common and uncommon heart diseases.

**Methods:**

A total of 156 subjects were imaged including 62 with normal findings, 33 patients with chronic myocardial infarction (MI), 33 with hypertrophic cardiomyopathy (HCM), 15 with non-ischemic dilated cardiomyopathy (DCM), 7 with acute myocarditis, 4 with cardiac amyloidosis, and 2 with systemic capillary leak syndrome (SCLS). Motion corrected ECV maps were generated automatically from T1-maps acquired pre- and post-contrast calibrated by blood hematocrit. Abnormally-elevated ECV was defined as >2SD from the mean ECV in individuals with normal findings. In HCM the size of regions of LGE was quantified as the region >2 SD from remote.

**Results:**

Mean ECV of 62 normal individuals was 25.4 ± 2.5% (m ± SD), normal range 20.4%-30.4%. Mean ECV within the core of chronic myocardial infarctions (without MVO) (N = 33) measured 68.5 ± 8.6% (p < 0.001 vs normal). In HCM, the extent of abnormally elevated ECV correlated to the extent of LGE (r = 0.72, p < 0.001) but had a systematically greater extent by ECV (mean difference 19 ± 7% of slice). Abnormally elevated ECV was identified in 4 of 16 patients with non-ischemic DCM (38.1 ± 1.9% (p < 0.001 vs normal) and LGE in the same slice appeared “normal” in 2 of these 4 patients. Mean ECV values in other disease entities ranged 32-60% for cardiac amyloidosis (N = 4), 40-41% for systemic capillary leak syndrome (N = 2), and 39-56% within abnormal regions affected by myocarditis (N = 7).

**Conclusions:**

ECV mapping appears promising to complement LGE imaging in cases of more homogenously diffuse disease. The ability to display ECV maps in units that are physiologically intuitive and may be interpreted on an absolute scale offers the potential for detection of diffuse disease and measurement of the extent and severity of abnormal regions.

## Background

Measurement of the extracellular volume fraction (ECV) in the myocardium could provide a quantitative method for assessing cardiomyopathies which may develop diffuse fibrosis or edema. Diffuse myocardial fibrosis is difficult to distinguish using late gadolinium enhancement (LGE) since the myocardial signal intensity is deliberately nulled to enhance the heterogeneity of abnormal myocardium thus homogeneously diffuse tissue will be nearly isointense and therefore globally “nulled” becoming indistinguishable from normal tissue [1]. ECV relates directly to the tissue distribution of most gadolinium-based contrast agents. A number of recent studies [1-8] have demonstrated the potential of ECV measurement. ECV maps are based on measurement of the longitudinal relaxation time constant (T1) both pre- and post-contrast, and are calibrated using the value of hematocrit [9].

Previous studies have measured the ECV using manual regions of interest drawn on T1-maps or by performing a manual or semi-automatic image registration of T1-maps. A fully automated method has been presented for calculating pixel-wise ECV parametric maps (ref Part I). The method included correction of respiratory and patient motion, and automatic segmentation.

The goal of this research was to evaluate the utility of the automated method for producing a pixel-wise map of ECV and to establish baseline quantitative values for normal subjects. We hypothesized that measurement of ECV, a physiological parameter directly related to the tissue distribution of an extracellular gadolinium-based contrast agent, would detect both focal and diffuse myocardial abnormalities in a variety of common and uncommon diseases. We established a normal range in 62 subjects. Next, we tested the hypothesis that patients with known regional abnormalities in the heart would have regional abnormalities in ECV by imaging patients with chronic myocardial infarctions and hypertrophic cardiomyopathy. Finally, we assessed the myocardial ECV in patients with diseases that might manifest with more diffuse abnormalities such as amyloidosis, non-ischemic dilated cardiomyopathy (DCM), and systemic capillary leak syndrome (SCLS).

## Methods

### Human subject protocol

ECV imaging was performed between Sept 2009 and May 2011. This study was approved by the local Institutional Review Boards of the National Heart, Lung, and Blood Institute and Suburban Hospital, and all subjects gave written informed consent to participate. Hematocrit was measured from a venous blood sample drawn just prior to the CMR study.

All patients were referred for CMR assessment of known or suspected heart disease prospectively underwent ECV imaging. For the purpose of this study, subjects were included in this analysis based on the following criteria. Normal subjects (n = 62) were used to establish baseline values of myocardial ECV. Normal subjects were defined as patients who were without known significant systemic illness, whose studies demonstrated normal biventricular systolic function, chamber size, and wall thickness, absent significant valvular dysfunction, and lack of late gadolinium enhancement. Subjects with hypertension or diabetes were excluded from this group and their clinical characteristics have been described (ref Part I). Chronic myocardial infarction (MI) was defined as a region of increased signal intensity on LGE that included the subendocardium that was within a coronary territory, and occurring in a patient with a clinical syndrome consistent with an acute coronary syndrome at least 6 months prior to the scan. Myocarditis was defined as a clinical syndrome established by the presence abnormal cardiac biomarkers (troponin I assay) in the absence of coronary artery disease, with a regional wall motion abnormality and increased signal intensity in the corresponding region on T2-weighted images. The diagnosis of hypertrophic cardiomyopathy (HCM) was supported by left ventricular hypertrophy (wall thickness >15 mm) in the absence of a clinical condition known to cause hypertrophy. If wall thickness was 10-15 mm, one or more additional criteria were required: (1) hypertrophy in a recognizable pattern like that of apical-variant HCM; (2) systolic anterior motion of the mitral valve with mitral regurgitation; and (3) resting left ventricular outflow tract obstruction. Cardiac amyloidosis was based on biopsy evidence of amyloidosis or presence of a disease known to cause cardiac amyloidosis (e.g. multiple myeloma). Nonischemic dilated cardiomyopathy (DCM) was defined as globally reduced systolic function (ejection fraction <45%) with an enlarged left ventricular chamber in the absence of obstructive coronary artery disease. Chronic systemic capillary leak syndrome (SCLS) was diagnosed according to established criteria (persistent peripheral edema and hypoalbuminemia in the absence of secondary causes) [10].

### Imaging

ECV maps were derived from T1-maps acquired pre- and post-contrast using the MOLLI method [11] calibrated by blood hematocrit as described previously (ref Part I). The approach incorporates correction of respiratory motion that occurs due to insufficient breath-holding [12] and due to patient movement between breath-holds. Additionally, cine MRI of cardiac function and phase sensitive inversion recovery (PSIR) late gadolinium enhancement (LGE) imaging [13] was performed on all subjects.

### Image and statistical analysis

The myocardium was manually segmented for ECV and LGE measurements. In subjects with regions of abnormal ECV, mean values of these regions were measured. In cases of chronic MI, ECV values were measured in the central core region of the MI.

The normal range of ECV values in the myocardium was defined as mean ± 2SD of ECV values in the normal group, and pixels were assessed as abnormal in LGE images for signal intensities greater than the mean + 2SD in a normal region in the same image.

Group statistics were measured for subjects with HCM (n = 33) and chronic MI (n = 33). ECV measurements were made on subjects with non-ischemic DCM (n = 15) and cardiac amyloidosis (n = 4) but group correlations to the size of LGE regions were not performed in these entities due to the small numbers and the diffuse nature of these processes.

The percentage of pixels with abnormally elevated ECV (> 30.4%) was compared with the percentage of enhanced pixels in LGE images that were greater than mean + 2SD measured in a remote region that appeared “normal”.

Data are presented as mean and standard deviation. Differences between means were tested by the paired or unpaired *t*-test, as appropriate. Statistical significance was defined as p < 0.05.

## Results

### Demographics

Patient characteristics for each group are summarized in Tables1 and 2. The self described race/ethnicity of the 156 subjects was as follows: 117 white, 20 black or African American, 14 Asian, and 5 Hispanic.

**Table 1:** Patient characteristics

			**Gender**			
	**N**	**Age (years)**	**Male**	**Female**	**BMI (m**^**2**^**)**	**Creatinine (mg/dL)**
		**(Mean+/−SD)**				**(Mean+/−SD)**
					**(Mean+/−SD)**	
Myocarditis	7	36.3+/−14.8	5	2	25.5+/−4.5	0.8+/−0.1
Nonischemic DCM	15	51.2+/−15.6	10	5	27.1+/−8.4	1+/−0.2
SCLS	2	53+/−11	0	2	35.5+/−6.2	1+/−0.2
Cardiac Amyloidosis	4	69+/−3.1	3	1	26.9+/−3.2	1.5+/−0.3
HCM	33	50.9+/−13.9	24	9	28.7+/−5.7	1+/−0.2
Chronic MI	33	57.3+/−9.5	27	6	25.2+/−5.7	1+/−0.2
Normal	62	43.6+/−17.4	30	32	26.5+/−4.6	0.9+/−0.2
	156		99	57		

**Table 2:** Patient characteristics (CMR measurements)

		**Left ventricular masses and volumes (Mean +/-SD)**					
	**N**	**LVEF (%)**	**LVEDV (mL)**	**LVEDVi (mL/m**^**2**^**)**	**LV mass (g)**	**LV mass indexed (g/m**^**2**^**)**	Wall thickness*
							**(mm)**
Myocarditis	7	56.2	167.2	89.6	106.5	57.2	8.2
		+/−4.8	+/−26	+/−13.3	+/−20.9	+/−11.7	+/−2.2
Nonischemic DCM	15	41.2	226.7	103.5	145.4	66.7	8.9
		+/−8.9	+/−61.9	+/−15.4	+/−42.8	+/−15.2	+/−2
SCLS	2	72.2	145.3	75.7	79.5	42.2	6.7
		+/−4.7	+/−17.8	+/−0.2	+/−2.5	+/−6.6	+/−0.4
Cardiac Amyloidosis	4	48.8	139	71.1	146.8	75.6	12.4
		+/−12.8	+/−45.4	+/−19	+/−44.3	+/−18.6	+/−1.8
HCM	33	61.8	163	83.8	169.4	86.7	19.1
		+/−5.4	+/−33.1	+/−13.2	+/−73.2	+/−36.1	+/−6.2*
Chronic MI	33	46.8	195.4	103.9	112.3	59.5	8.8
		+/−14.2	+/−62	+/−33.9	+/−28.6	+/−14.8	+/−1.9
Normal	62	61.7	143.9	76.9	87.4	46.2	7.5
		+/−5.9	+/−32.9	+/−12.2	+/−26.2	+/−9.5	+/−1.8
	156						

BMI = Body Mass Index, DCM = Dilated Cardiomyopathy, SCLS = Systemic Capillary Leak Syndrome, HCM = Hypertrophic Cardiomyopathy, MI = Myocardial Infarction, LV = left ventricular, EF = ejection fraction, EDV = end-diastolic volume. Wall thickness was measured in the basal anteroseptum in all conditions except HCM where wall thickness represents maximal diastolic wall thickness in any short axis slice.

#### ECV measurements in normal subjects

**Figure 1:**
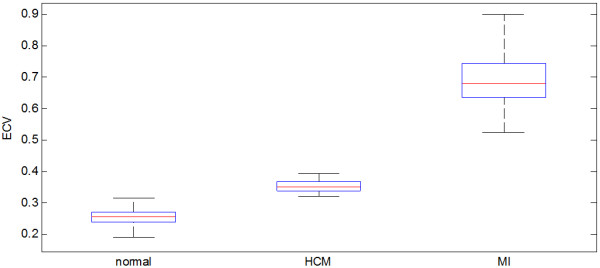
**ECV values for normal myocardium (n = 62), focal abnormalities in HCM (n = 33), and chronic MI (n = 33).** Box and whisker plots show median, 25 and 75 percentiles, and range.

ECV measurements (Figure 1) were made in 62 normal subjects that were LGE negative and appeared clinically normal during independent clinical interpretation. Mean ECV in normal individuals was 25.4 ± 2.5% (m ± SD) yielding a normal range for myocardial ECV of 20.4-30.4%. The pre-contrast T1 was 964.6 ± 35.3 ms yielding a normal range 894 to 1035 ms.

### ECV measurements in abnormal studies

**Figure 2:**
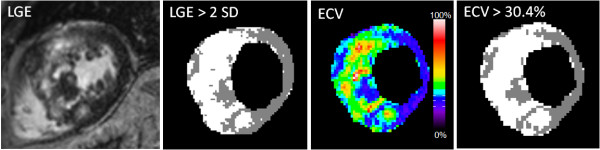
**LGE and corresponding ECV map for subject with HCM.** LGE values > mean + 2SD measured in a “normal” region and ECV values > 30.4% fixed threshold (corresponding to the mean + 2SD for myocardium in a normal group) are classified as abnormal.

**Figure 3:**
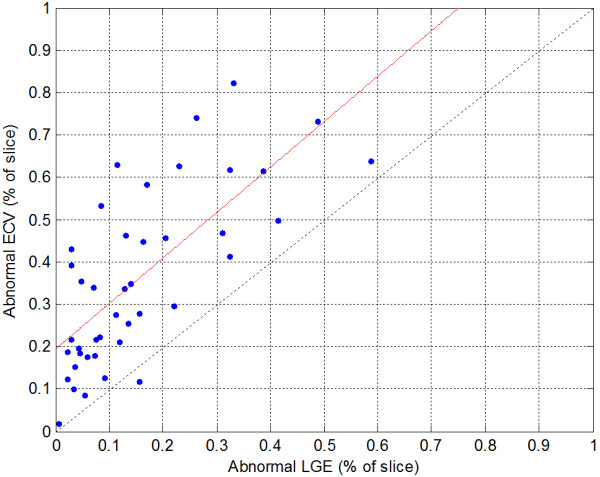
**Comparison of the percent of myocardium classified as abnormal by ECV using a fixed threshold (ECV > 30.4%) and the percent of LGE > mean + 2SD (measured in remote “normal”).** Linear fit (red) is%ECV = 1.07%LGE = 0.19.

In the 33 patients with HCM, the percentage of myocardium with elevated ECV correlated with the amount of myocardium with abnormal LGE (R = 0.72, p < 0.001) (Figure 2) and the amount of myocardium with abnormal ECV systematically exceeded the amount of myocardium with abnormal LGE (% ECV = 1.07% LGE + 0.19, Figure 3). HCM subjects had mean ECV values for pixels above the normal range (Figure 1) equal to 35.7 ± 2.9% (m ± SD) (p < 0.001 vs normal). The mean ECV value of “normal” pixels was 26.5 ± 1.3%. Myocardial ECV values in HCM were more heterogeneous and had lower mean value than for MI (p < 0.001, Figure 1).

ECV values for chronic MI (n = 33) were equal to 68.5 ± 8.6% (p < 0.001 vs normal). There were no cases of chronic MI with microvascular obstruction.

In patients with acute myocarditis (n = 7), all subjects had focally elevated ECV > 30.4%. ECV values for subjects with elevated ECV were 44 ± 6% and 26.4 ± 3%, in focally elevated and remote regions, respectively.

There were 18 subjects with non-ischemic DCM, of whom 3 did not have measurable ECV due to patient movement between pre- and post-contrast acquisitions. Regions of abnormally elevated ECV were identified in 4 of 16 patients with non-ischemic DCM and LGE in the same slice appeared “normal” in 2 of these 4 patients. ECV was 38.1 ± 1.9% (p < 0.001 vs normal) in the non-ischemic DCM patients with elevated ECV.

There were 4 subjects with cardiac amyloidosis. The ECV values for these subjects had mean values ranging from 32 to 60% with an overall mean of 46 ± 12%.

In subjects with SCLS with abnormally elevated ECV (n = 2), the ECV had a mean ECV of 41% with global distribution throughout the heart. Furthermore, the mean pre-contrast T1 was 1145 ms, which is abnormally long.

#### ECV maps in diseases with focal gadolinium enhancement

**Figure 4:**
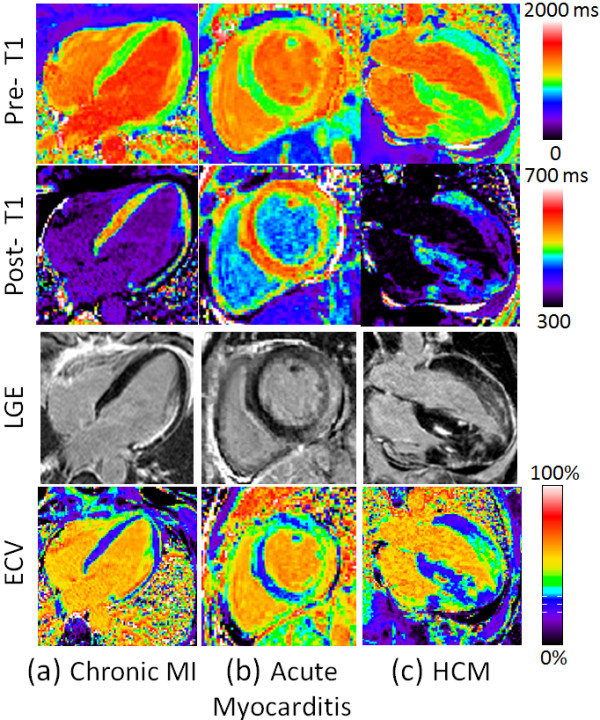
**Examples illustrating excellent agreement between LGE and ECV in cases of focal abnormalities in myocardial ECV.** Pre-contrast T1-maps (top row), post-contrast T1-maps (2^nd^ row), late gadolinium enhancement (3^rd^ row), and ECV maps (bottom row) for patients with: (**a**) chronic MI, (**b**) acute myocarditis, and (**c**) HCM.

ECV maps, T1-maps pre- and post-contrast, and corresponding PSIR late gadolinium enhancement images are shown in Figure 4 as examples of focal enhancement including (a) chronic MI, (b) myocarditis, and (c) HCM. Focal scar is readily detectable in both the conventional late gadolinium enhancement and ECV maps with excellent spatial agreement. In these figures, ECV maps and T1-maps are displayed with a fixed window-level (ECV from 0-100%, pre-contrast T1 from 0 to 2000 ms, and post-contrast T1 from 300–700 ms), while PSIR LGE images are displayed to null normal myocardium.

Note that the post-contrast T1-maps have a considerably different appearance using a fixed scale due to the variation of renal clearance of gadolinium, body composition and baseline T1, further emphasizing the difficulty of directly using post-contrast T1 values alone without ECV calculation. Elevated pre-contrast T1 is pronounced in the subject with acute myocarditis (Figure 4(b)), which is expected in a patient with myocardial edema. For this specific case of acute myocarditis the region corresponding to signal enhancement on LGE had measured values of T1_pre_/T1_post_/ECV = 1249 ms/ 487 ms / 51%.

### ECV maps in diseases with diffuse gadolinium enhancement

**Figure 5:**
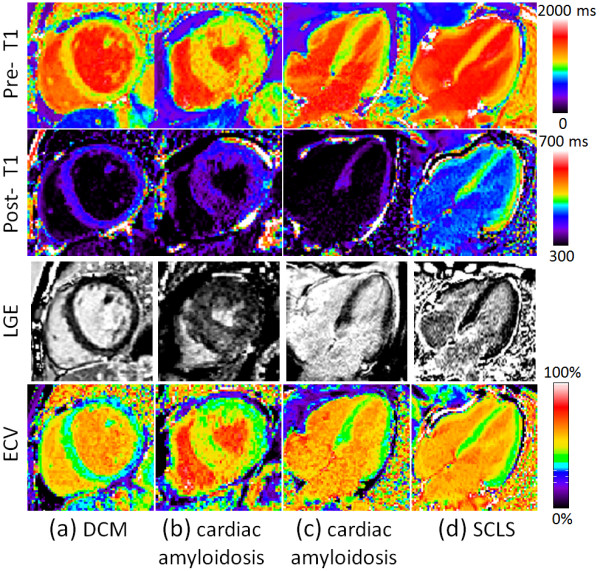
**Examples illustrating cases with diffuse abnormalities in myocardial ECV which are challenging to assess with conventional LGE.** Pre-contrast T1-maps (top row), post-contrast T1-maps (2^nd^ row), late gadolinium enhancement (3^rd^ row), and ECV maps (bottom row) for patients with various cardiomyopathies: (**a**) non-ischemic DCM with diffusely elevated ECV and normal appearing LGE, (**b**) cardiac amyloidosis with “patchy” LGE enhancement, (**c**) cardiac amyloidosis with diffusely elevated ECV and globally nulled LGE, and (**d**) systemic capillary leak syndrome (SCLS) with globally elevated ECV due to edema and normal LGE.

Figure 5 contains examples with diffusely elevated ECV for subjects with (a) non-ischemic dilated cardiomyopathy (DCM), (b, c) cardiac amyloidosis, and (d) systemic capillary leak syndrome (SCLS) [10]. In these more diffuse cases it is difficult to distinguish abnormal from normal regions using conventional late enhancement. Notably, the ECV image provides quantitative characterization of myocardium in physiologically intuitive units of measurement. In 3 of these cases (Figure 5(a, c, d), the LGE is nulled reasonably well on a global basis, thereby appearing normal.

For the DCM case (Figure 5(a)) values were T1_pre_/T1_post_/ECV = 1103 ms/ 387 ms/ 37% in the septum and 1027 ms/426 ms/30% in the inferior wall. For the subject with cardiac amyloidois (Figure 5(c)) measured values were T1_pre_/T1_post_/ECV = 1028 ms/356 ms/ 41%. For the subject with SCLS (Figure 5(d)) measured values were T1_pre_/T1_post_/ECV = 1126 ms/496 ms/41%. In one subject with cardiac amyloidosis (Figure 5(b)), the LGE image is diffusely enhanced in the septal region but might be interpreted as normal in the inferior wall which may be nulled. For this case (Figure 5(b)) measured values were T1_pre_/T1_post_/ECV = 1139 ms/351 ms/51% in the septum and 1059 ms/394 ms/41% in the inferior wall (i.e., the inferior wall appears normal by LGE but has abnormally elevated ECV).

## Discussion

The main finding of the study is that ECV-mapping can quantitatively characterize myocardial tissue characteristics over a broad range of myocardial diseases including pathophysiological states with either focal and/or diffuse changes in myocardial ECV. In cases of focal scar such as chronic MI and focal edema and/or necrosis such as acute myocarditis, ECV maps were in excellent agreement with LGE. These cases were measured to provide a confirmation that the ECV performs accurately in instances where the resulting enhancement is known and readily assessable. In cases with patchy LGE such as HCM, the LGE is sufficiently heterogeneous that it is possible to null normal appearing regions of myocardium thus providing a baseline for detection of patches with late enhancement. In these cases, the regions of elevated ECV correlated well with the regions of LGE. In cases of HCM, it was possible to detect abnormal tissue regions using a fixed threshold derived from statistics of the normal group independent of the presence of normal appearing regions within the slice of interest.

The current study presents quantitative myocardial ECV measurements in the settings of chronic myocardial infarction, HCM, DCM, myocarditis, amyloidosis and SCLS. Previous studies have measured myocardial ECV in aging [6,7], chronic myocardial infarction [7,14], non-ischemic cardiomyopathies in general [7] and in particular DCM [3], aortic stenosis and HCM [2] and congenital heart disease [5]. Our current results broadly agree with these prior reports with regards to the magnitude of ECV in both focal and diffuse myocardial disease. With the exception of Ugander, et al. [7], all previous reports are based on region of interest measurements performed in images that do not permit visualization of the spatial extent of variations in myocardial ECV. The methods of Ugander, et al., [7] required time consuming and cumbersome manual post-processing for motion correction. The current work, as a continuation of those findings, broadens the clinical findings and presents results using a method for fully automated generation of ECV images in a clinically feasible work flow.

As described previously (ref Part I), there are cases in which patient movement resulted in the slices being substantially different in appearance despite the fact that the same slice position was prescribed. Co-registration is by design only capable of correcting for in-plane motion and cannot compensate for through plane motion, although it may appear to partially compensate. In other cases, it is clear that the pre- and post-contrast images are at substantially different cardiac phases due to significant change in heart rate, despite adjusting the trigger window. Both of these factors could potentially be improved at the time of acquisition by more careful inspection at the cost of a more complicated workflow.

The ability to interpret lower quality ECV maps and not mistake uncorrected motion artifacts or partial volume border pixels for sub-endocardial or sub-epicardial fibrosis is a residual and important issue. Motion related errors occur both intra-series and between pre-and post-contrast series. The correction of intra-series motion due to inadequate breath-holding was referred to as MOCO, whereas the correction of subject motion between pre- and post-contrast acquisition was referred to as co-registration. The use of error maps based on residual errors in pixel-wise T1-fitting provide a means of determining the MOCO error, and may serve as a quality metric. In terms of co-registration errors, the interpretation can be improved by examining the pre-and post-contrast T1-maps for consistent appearance of position and cardiac phase as a quality control step. Furthermore, it is expected that the use of ECV maps will be in conjunction with pre-contrast T1-maps and conventional late gadolinium enhancement images, and not interpreted independently. As such, the ability to assess and quantify diffuse fibrosis in ECV images should provide increased diagnostic confidence since LGE image quality is generally excellent and will not share the same artifacts.

The partial volume issue is not only important in terms of the visual readout, but may introduce biases into quantitative measurements which become particularly significant in the context of more subtle diseases. These effects are less problematic for globally diffuse processes. One approach to reducing the bias in measurements is to restrict the measurement to the mid-wall region, although one must exercise caution for subjects with thin walled myocardium or patients with thin rims of subendocardial fibrosis. Blood pool contamination of the myocardium in border pixels can lead to biases if included in the measurement, and in general may contribute more significantly in subjects with thin myocardial wall thickness. This might lead to an apparent elevated ECV in subjects with thin walls such as healthy females or subjects with DCM, and thereby might lead to an underestimation of the difference in these populations with subjects that have hypertrophy since thicker myocardium should have less error due to partial volume at the borders.

Quantitative ECV pixel maps may have potential for analysis of border zones around MI. However, caution must be exercised in differentiating partial volume effects from true pathophysiological mechanisms. Partial volume effects affect the measurement of ECV by contributing to larger variability in fine structures such as subendocardial MI. Similar to LGE, the partial volume “blurring” due to the slice thickness may contribute significantly to the apparent border zone.

Despite these issues, ECV maps may be readily incorporated into the clinical workflow and may provide diagnostic information where other methods are limited. It is not intended to replace existing methods such as LGE which are excellent at depicting focal lesions, but rather to be used in concert with other techniques. When ECV is abnormally elevated, it may not be clear whether this is due to fibrosis or edema which may be either diffuse or focal. In such instances, pre-contrast T1 or T2 maps [15], in addition to patient history and contextual imaging clues like signs of heart failure, may help to differentiate these mechanisms. Finally, several cases were presented for subjects with diffusely elevated LGE. These demonstrate the proposed utility of ECV mapping in cases which are frequently challenging to assess.

### Limitations

The number of subjects is small for some of the subgroups in this study. In these cases, large scale studies are needed to establish the clinical utility. In this study which compared the percentage of abnormal tissue by ECV measurement and LGE using mean + 2SD threshold, it was not possible to conclude which measurement was more accurate. Estimates of both the mean and SD in normal appearing LGE regions are generally subject to being biased on the high side due to signal inhomogeneities, and in this case could explain the underestimation by LGE using this type of thresholding.

## Conclusions

ECV mapping appears promising to complement LGE imaging in cases of more homogenously diffuse myocardial disease states which affect the myocardial extracellular space. The ability to display ECV maps in quantitative and physiologically intuitive units that may be interpreted on an absolute scale offers the potential for simplified detection and measurement of the extent and severity of abnormalities affecting the myocardial ECV.

## Abbreviations

CMR, Cardiovascular Magnetic Resonance; DCM, Dilated cardiomyopathy; ECV, Extracellular volume fraction; HCM, Hypertrophic cardiomyopathy; LGE, Late gadolinium enhancement; MI, Myocardial infarction; MOCO, Motion correction; MOLLI, Modified Look-Locker inversion recovery; SCLS, Systemic capillary leak syndrome; TI, Inversion time; ROI, Region-of-interest; PSIR, Phase sensitive inversion recovery.

## Competing interests

Dr. Arai is a principal investigator on a US government Cooperative Research And Development Agreement (CRADA) with Siemens Medical Solutions (HL-CR-05-004).

## Authors’ contributions

PK participated in design of the study, developed the technical approach, algorithms and software, performed processing and analysis, and drafted the manuscript. JRW performed analysis of clinical studies and diagnoses. XH developed software used for motion correction. JRW and MU participated in design of the study. WPB and SMS contributed to clinical diagnosis, and KMD contributed his expertise in subjects with SCLS. AEA conceived of the study and participated in analysis. All authors contributed to editing of the manuscript and read and approved the final manuscript.
